# Food Outlets Dietary Risk (FODR) assessment tool: study protocol for assessing the public health nutrition risks of community food environments

**DOI:** 10.1186/s12937-020-00641-w

**Published:** 2020-11-12

**Authors:** Claire Elizabeth Pulker, Georgina S. A. Trapp, Mark Fallows, Paula Hooper, Heather McKee, Christina Mary Pollard

**Affiliations:** 1 School of Public Health, Curtin University, Kent Street, GPO Box U1987, Perth, Western Australia 6845 Australia; 2 East Metropolitan Health Service, Kirkman House, 20 Murray Street, East Perth, Perth, Western Australia 6004 Australia; 3 Telethon Kids Institute, The University of Western Australia, PO Box 855, West Perth, Western Australia 6872 Australia; 4 School of Population and Global Health, The University of Western Australia, 35 Stirling Highway, Crawley, Western Australia 6009 Australia; 5 Department of Health of Western Australia, Perth, Western Australia 6004 Australia

**Keywords:** Community food environments, Food outlet, Food retail, Supermarket, Fast food outlet, Local government, Risk assessment, Diet

## Abstract

**Background:**

Availability and accessibility of nutritious foods can vary according to the food outlets present within a neighbourhood or community. There is increasing evidence that community food environments influence food choice, diet and the risk of diet-related chronic disease, however contemporary community food environments assessments (e.g. unhealthy fast food outlets versus healthy supermarkets or fruit and vegetable shops) may be too simplistic to accurately summarise the complexities of their impacts on food choice. This study protocol describes the development of the Food Outlets Dietary Risk (FODR) assessment tool for use by local government in Perth, Western Australia.

**Methods:**

Similar to food safety risk assessment, the FODR assessment tool rates the potential harmful public health nutrition impact of food outlets by identifying and characterising the issues, and assessing the risk of exposure. Scores are attributed to six public health nutrition attributes: 1) availability of nutrient-poor foods; 2) availability of nutritious foods; 3) acceptability and appeal; 4) accessibility; 5) type of business operation; and 6) complex food outlet considerations. Food retail outlets are then classified as having a low, medium, high or very high dietary risk based on their total score.

**Discussion:**

A local government administered tool to rate the public health nutrition risk of food outlets requires data which can be collected during routine assessments or sourced from the internet. The ongoing categorical classification of foods available within food outlets as either unhealthy or nutritious will require nutrition scientists’ input. An objective risk assessment of the dietary impact of food retail outlets can guide local government planning, policies and interventions to create supportive community food environments. It is intended that locally relevant data can be sourced throughout Australia and in other countries to apply the local context to the FODR assessment tool. Utility and acceptability of the tool will be tested, and consultation with environmental health officers and public health practitioners will inform future iterations.

**Supplementary Information:**

The online version contains supplementary material available at 10.1186/s12937-020-00641-w.

## Background

There is increasing evidence that food environments influence food choices and diet, therefore improving food environments to reduce the risk of diet-related chronic disease is a public health priority [1–3]. The availability and accessibility of healthy food is an important contributor to healthy diets [4], and varies according to the food outlets present within a neighbourhood or community [3]. The ability of community food (or nutrition) environments to support healthy eating can be measured by identifying the number, type, location and accessibility of food outlets within a specified area [5]. Governments, non-government health organisations and researchers increasingly use food outlet listings to attempt to assess the relative healthfulness of community food environments [6]. Two commonly used measures are proximity to food outlets, and total count or density of food outlets within a neighbourhood [7].

To date, community food environments assessment has mainly focused on identifying and mapping a limited number of food outlet types using supermarkets or greengrocers as a proxy for healthy food access, and fast food or takeaway outlets as a proxy for unhealthy food access [8, 9]. For example, the United States (US) Center for Disease Control and Prevention’s modified Retail Food Environment Index (mRFEI) is a ratio of outlets considered to be healthy (supermarkets, large grocery stores, supercentres, and greengrocers) to outlets considered to be less healthy (fast food, small grocery stores, and convenience stores) [10]. Whilst there is increasing evidence that food environments contribute to food choices and diet [1, 2], evidence of an association between community food environments and diet related non-communicable diseases is inconsistent [11, 12]. Existing classification and measurement approaches may be too simplistic to accurately summarise the complexities of food outlet impacts on food choice [13].

The role supermarkets play within community food environments is unclear [9]. Australian supermarkets and grocery stores are an important source of healthy foods but they are also the main retailer of unhealthy (discretionary) foods that government dietary advice recommends to limit consumption of [14]. For example, in Australia 57% of total soft drinks sales and 48% of total snack foods sales were from supermarkets in 2017 [15]; and less than half of supermarket packaged foods are classified as healthy [16]. Most Australian supermarket checkouts display soft drinks and unhealthy snack foods including crisps, chocolate, and confectionery [17–19] and 74% of Australians purchase alcohol from a supermarket or stand-alone store owned by a supermarket group [20]. Shopping primarily at supermarkets was not associated with a better nutrient profile of packaged foods purchased in the US [21]; and in fact, despite supermarkets being the main retailer of food for the population (ensuring food security), few positive public health impacts of Australian supermarkets have been identified [22].

Some anomalies also exist in fast food outlets and takeaways. Studies conducted in the United States (US) and United Kingdom (UK) indicate fast food is energy-dense and nutrient-poor compared to home prepared food [23, 24], and there is an association between growth in fast food consumption and obesity in Australia [25]. Burgers are the main products sold at Australian fast food outlets (31%), followed by pizza (27%), and chicken-based fast food (18%) [26]. Australian fast food or takeaway outlets can also provide healthful options such as fresh salad, wholemeal or wholegrain bread, dishes with a high proportion of vegetables, dishes that are not deep-fried, and fresh fruit [27, 28]. Therefore, a more nuanced system to rate the risk of food outlets to public health nutrition is needed to assist with understanding and potentially modifying the impact of community food environments on population diets.

Two Australian studies which developed detailed classifications of healthy and unhealthy food outlets have recognised these limitations [8, 29]. Thornton and Kavanagh (2012) assigned ratings from + 10 (most healthy) to − 10 (least healthy) to 20 types of food outlets, based on consultation with eight Australian and international academic researchers [8]. The participants were selected on the basis of conducting food environments research, and were asked to consider four factors when assigning their scores. Moayyed et al. (2017) also sought expert opinion from 26 Australian public health researchers and practitioners on the relative healthfulness of 24 food outlet types using a Delphi survey to rate the outlets from + 10 (most healthy) to − 10 (least healthy) [29]. The participants were given a proposed set of scores for the outlets to comment on in two rounds of surveys; however, it is unclear how the proposed scores were derived or the factors considered by participants when making comments. In summary, the criteria that informed the ratings attributed in these studies were not transparent or consistent.

The commonly used risk assessment approach, adopted by the Codex Alimentarius system of international food standards [30, 31] comprises science-based identification, characterisation and analysis of issues, to estimate the likelihood and severity of an adverse health event occurring [32] may be a useful approach. Australia’s Food Standards Australian New Zealand (FSANZ) applies a risk analysis approach which includes risk assessment, risk management and risk communication [32]. FSANZ has predominantly focussed on food safety as the priority to protect public health and safety; however the risk analysis approach could also be used to improve public health nutrition [33]. In Western Australia (WA), the Department of Health works in partnership with local government agencies (LGAs) to protect environmental health with a particular focus on food safety. The State government’s Department of Health oversees the food regulatory system while LGAs are responsible for monitoring food standards within their district [34]. LGAs routinely assess food businesses as part of their food safety surveillance system but the relative healthfulness of the foods sold and potential impact on diet and health does not form part of this assessment. WA LGAs also have statutory responsibility for protecting community health and wellbeing in accordance with the *Public Health Act 2016* [35]. Public health plans describing the strategies to be implemented to meet community public health needs are required from each LGA, which includes creating and maintaining healthy environments [35]. The risk assessment approach routinely used by LGAs for food safety could also be applied to public health nutrition risk to identify the relative healthfulness of food outlets and their potential impact on diet related non-communicable diseases. A tool to assist LGAs to rate the potentially harmful public health nutrition impact of food outlets is therefore needed.

## Methods/design

### Study aim

The aim of this protocol is to describe development of the Food Outlet Dietary Risk (FODR) assessment tool which incorporates public health nutrition issues. The FODR assessment tool is intended to be used to rate the potentially harmful public health nutrition impact of individual food outlets (i.e. food businesses with a consumer-facing retail or food service component), to inform surveillance and planning.

Public health nutrition includes the determinants of health that relate to a safe, nutritious, affordable, accessible, secure and environmentally sustainable food system [36]. Environmental sustainability is outside of the scope of this protocol because there are no recommendations or measures for environmental sustainability of the Australian population diet.

The risk assessment principles applied to the protocol include: using the best available evidence, recognising the inherent uncertainty in risk assessment, and using an iterative process [32].

### Setting

The WA Department of Health delivers health services to the Perth metropolitan population via three area health services, including East Metropolitan Health Service (EMHS). EMHS provides tertiary, secondary, specialist, and community health care services that aim to maintain and improve the health of one-third of the WA population [37]. Thirteen LGAs are located within the EMHS geographic area, and they provide the setting for the current research.

### Study design

The Standard Protocol Items: Recommendations for Interventions Trials (SPIRIT) checklist was used to guide this protocol (Supplementary Table 1) [38].

#### Compilation of a database of food businesses

LGAs are responsible for registering food businesses in WA. EMHS staff contacted the environmental health team at each of the 13 LGAs in April 2018 to request a list of registered food businesses within their area. A formal letter was followed up by telephone calls to address any questions concerning use of the data, and all data were provided by June 2018. A database of 6963 registered food businesses present in the EMHS area was constructed, which included 4136 food outlets, i.e. businesses with a food service or food retail component which sells foods to consumers.

#### Classification of all food businesses

A classification framework that identifies consumer-facing food retail (e.g. supermarkets, greengrocers) and food service outlets (e.g. cafes, takeaways), food preparation businesses (e.g. food manufacturers, wholesalers), charitable food provision (e.g. food pantries), and institutional food (e.g. school canteens, hospitals) was developed (Supplementary Table 2). Food outlets identified as consumer-facing are the focus of this study.

#### Risk assessment of food businesses

**Fig. 1:**
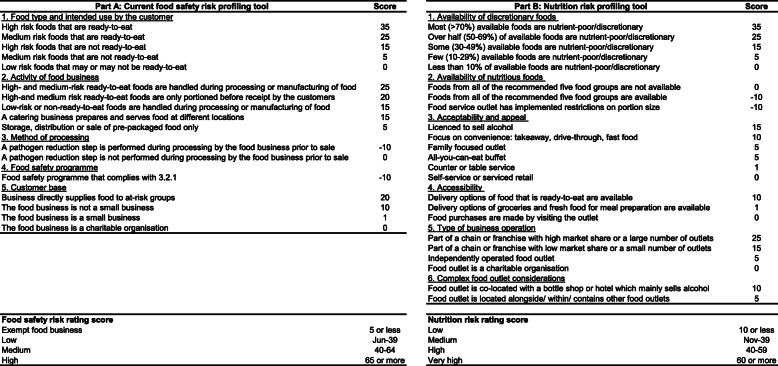
The Food Outlet Dietary Risk Assessment Tool^1^. Department of Health of Western Australia. WA food regulation: food business risk profiling. Guidance to the classification of food businesses. Australia: Government of Western Australia; 2016. Available from: https://ww2.health.wa.gov.au/~/media/Files/Corporate/general%20documents/food/PDF/WA_Food_Regulation_Food_Business_Risk_Profiling.pdf

The WA Department of Health provides guidance to LGAs on risk assessment of food safety, including a tool which rates the following: food type and intended consumer use, activity of the food business, method of processing, size of customer base, and implementation of a food safety programme [34]. To assist with translation, the FODR assessment tool replicated this approach. Part A of the tool duplicates the existing WA Department of Health guidance for assessing food safety risk; part B incorporates dietary risk assessment (Fig. 1).

#### Public health nutrition issue identification and characterisation

The public health nutrition issue being addressed is diet quality consistent with the Australian Dietary Guidelines and Australian Guide to Healthy Eating recommendations [39]. Characterisation of the issue was informed by the recommendations made in three of the Australian Dietary Guidelines [40]. Guideline one states “To achieve and maintain a healthy weight, be physically active and choose amounts of nutritious food and drinks to meet your energy needs”; guideline two recommends eating a wide variety of foods from the nutritious five food groups every day as well as drinking water; and guideline three refers to limiting intake of discretionary foods that contain saturated fat, added salt, added sugars and alcohol [40]. Few Australians meet the nutritious five food group recommendations, only 8 % of adults ate the recommended amount of vegetables and 5 % met recommendations for both fruit and vegetables in 2017–18 [41]. The 2011 National Nutrition Survey found that only 10 % of Australians ate enough dairy foods and alternatives; 14% ate enough lean meats and alternatives; and 30% ate enough cereal (grain) foods [42]. Nutrient-poor discretionary foods accounted for 35% of total dietary energy intake [42]. In terms of health risk, poor quality dietary patterns are responsible for one in five preventable deaths globally [43]. The typically poor quality Australian diet has contributed to an increased prevalence of non-communicable diseases [41]. Almost half of Australians have at least one chronic disease, and the proportion of Australian adults classified as overweight or obese increased from 63% in 2014–15 to 67% in 2017–18 [41].

Substantial changes in dietary patterns are needed at a population level to meet the recommendations of the Australian Dietary Guidelines and reduce dietary risk. The FODR assessment tool therefore assesses the risk associated with the availability of discretionary foods, which is offset by the availability of foods from all five food groups (Fig. 1). To reflect the importance of appropriate serving sizes on the amount of food consumed, portion control in food service outlets is also included as an offset to the risk score (Fig. 1). To inform the risk assessment related to availability of healthy and unhealthy food, a literature review was conducted and is summarised in Supplementary Table 3.

#### Other risks identified

The acceptability, appeal, and accessibility [40] of food outlets which sell discretionary foods further impact the likelihood and severity of poor nutritional quality population diets.

The acceptability or appeal of food outlets can be influenced by making foods more convenient for consumers. For example, licenced food outlets make it easy for diners to consume alcohol. Food outlets with a drive-through takeaway service provide consumers the convenience of ordering and collecting food without leaving their car. A food outlet that provides playgrounds, toys, or special children’s menus, aims to appeal to families with young children by making the experience enjoyable for the whole family. Food outlets with all-you-can-eat buffets can appeal to the budget conscious but encourage over eating. The FODR assessment tool rates the risk associated with the acceptability and appeal of food outlets, using inputs from information obtained from the internet or audits of food outlets (Fig. 1).

The risk attributable to accessibility considers the likelihood of impulse purchases, which are unplanned purchases. There is a risk associated with ordering ready-to-eat foods online or by telephone as purchase decisions can be impulsive; and unplanned purchases are often triggered by a desire to indulge [44]. Purchasing supermarket groceries online can also lead to less healthy choices [45], and marketing strategies including altering the order in which products are displayed can influence purchase decisions [46, 47]. The FODR assessment tool rates the risk associated with online and telephone ordering using inputs from information obtained from the internet or audits of food outlets (Fig. 1).

The risk attributable to exposure is assessed by rating the type of food business operation. Food outlets that are part of a chain or franchise typically have a stronger market position, and are likely to have greater exposure to the population. This exposure can be due to the visibility gained by establishing a large number of outlets, or via investing in advertising and promotion to build brand awareness and influence consumer food choices [48]. To rate this risk, market share of the food outlet was estimated using market reports, such as the IBISWorld series [26, 49–54] (see Supplementary Table 4).

The final risk that is incorporated into the FODR assessment tool focuses on the increase in impact achieved by co-locating food outlets within one area to increase overall appeal. For example, Australian food courts have a large number of food service outlets co-located around a shared seating area and are often found in indoor shopping centres. Some supermarkets are co-located with an off-premises alcohol retailer, and increased availability and accessibility of alcohol outlets can lead to greater acceptance of alcohol and increased consumption [55]. Inputs to the FODR assessment tool are from information obtained from the internet or audits of food outlets.

#### FODR assessment tool scoring system

The FODR assessment tool’s scoring system allocates more points to factors that pose a greater risk to public health nutrition, and food outlets are classified as having low, medium, high or very high risks depending on the overall score achieved. Development of the scoring system took into consideration the public health goal of improving the impact of food outlets on community food environments, signalling ways food outlets can improve their score. The scoring system allocates the most points to food outlets where most available foods are discretionary; therefore a food outlet could reduce their risk score by introducing more recommended nutritious foods and reducing the proportion of discretionary foods. Chains or franchises with a dominant market position are also scored a high number of points, which reflects their level of influence over food selection.

#### Pilot testing

To evaluate the utility and acceptability of the FODR assessment tool, food outlet data provided by one of the 13 LGAs within the EMHS area will be pilot tested by the authors. A database will be created that guides the data entry process, with prepopulated fields of data where appropriate (for example the availability of discretionary and nutritious foods in supermarkets). The ability to source data and the practical effort required will be assessed. The scores generated by the FODR assessment tool for all food outlets present in the selected community food environment will be spatially mapped using Geographical Information System (GIS) technology. Statistical analysis will be conducted including: an average risk assessment score, number of food outlets scoring high or very high public health nutrition risks, and kernel density analysis of all food outlets (which estimates the density of referenced points rather than the proximity). Interested and affected groups, including LGA environmental health officers and public health practitioners, will be consulted and further analyses identified during the consultations will be incorporated into the pilot testing phase. Results from pilot testing, and input received during consultations, will inform future iterations of the FODR assessment tool. Validation of the risk rating scores will be conducted, to identify whether the scores allocated by the FODR tool accurately indicate the community exposure to public health nutrition risk.

## Discussion

The aim of this protocol was to describe development of a food outlets risk assessment tool which can be used to rate their public health nutrition impact. The FODR assessment tool was developed to assist LGAs within the EMHS area to identify food outlets that have a high or very risk to public health nutrition, by replicating the approach recommended by the WA Department of Health for food safety risk assessment. The risk is rated by inputting data to the tool, to provide an objective assessment of the food outlet’s impact on food safety (part A) and public health nutrition (part B). The tool is suitable for use throughout Australia, and could also be adapted for use in other countries.

The FODR assessment tool requires robust data for six public health nutrition attributes for each food outlet: 1) availability of discretionary foods; 2) availability of nutritious food; 3) acceptability and appeal; 4) accessibility; 5) type of business operation; and 6) complex food outlet considerations. For food safety risk assessment, data is usually obtained during the new business registration process with LGAs, and environmental health officer assessments of food outlets. Additional data related to rating the public health nutrition risk could also be collected during these processes, including whether the food outlet is licensed to sell alcohol, offers a takeaway or drive-through service, provides children’s toys or a play area, provides an all-you-can-eat buffet, delivers foods (including via a delivery service such as Uber Eats), or is co-located with a bottle shop or within a food court. Routine LGA data collected from registered food businesses could also capture whether the food outlet is independently operated or part of a franchise or chain, and details of the parent company. However, classification of available foods according to the recommendations of the Australian Dietary Guidelines requires nutrition or dietetics expertise, and it is not practical to advise environmental health officers to assess all products in each food outlet to determine the proportions of discretionary and nutritious foods. Published peer-reviewed surveys which describe the availability of discretionary and nutritious foods in different types of food outlets can be used instead, where available. For example, an audit of Australian supermarket packaged foods (not fresh foods) found that half were classified as discretionary [16], which can be applied to the risk assessment of all supermarkets.

An alternative but more time-intensive method to determine availability of discretionary and nutritious foods is to conduct audits of the food outlets using a validated tool such as the Nutrition Environment Measures Survey for supermarkets or restaurants (NEMS-S and NEMS-R [56, 57]) which were developed to assess the healthfulness of food outlets in the US and have also been used in Australia [58, 59], Brazil [60], Malta [61] and Canada [62]. The NEMS scores the availability, price and quality of a small number of healthier and less healthy foods, selected on the basis of their importance to population diets. To assist with completing the dietary risk assessment, a similar measurement tool could be developed to indicate the availability of discretionary foods and nutritious foods, for use by LGA environmental health officers during routine food business assessments. It is important to note that validation studies will be needed to ensure any new tools developed are credible, reliable and fit-for-purpose.

Frequency of food outlet assessment of dietary risk can be pragmatically guided by established processes for monitoring food safety risk by local government environmental health officers. In WA, the Department of Health recommends an approach which considers the risk profile of each food business (refer to Fig. 1, Part A). The frequency of food safety audit is guided by the risk rating score, with targets of assessing high risk food businesses every 3–12 months, medium risk food businesses every 6–18 months, and low risk food businesses every 12–24 months [34].

To improve the healthfulness of community food environments, LGAs will need to develop appropriate policies and interventions based on the dietary risk assessment of food outlets. In WA, the requirements of the Public Health Act provide a legal mechanism for encouraging action by LGAs. The FODR tool could be used to establish baseline scores for all food outlets in an LGA’s geographic area, with actions to improve ratings provided in public health plans. The ‘Nourishing’ framework of food policies to promote healthy diets and reduce non-communicable diseases recommends setting incentives and rules to create healthy food environments, for example setting limits on the number of fast food or takeaway outlets allowed near schools [63]. However, only 15 UK LGAs and one US city have registered the action they’ve taken to restrict unhealthy food outlets with the Nourishing database [64]. A recent review identified that 17% of surveyed UK LGAs had implemented health focused planning restrictions to limit hot food takeaways including: setting exclusion zones around schools, parks, sports centres, and youth clubs; restricting opening hours during school lunch breaks and immediately after school; setting limits to the density of takeaway food outlets permitted in retail areas; setting community infrastructure levies with funds raised being used for obesity prevention; mandating participation in healthy catering schemes; and requiring health impact assessments to accompany planning applications for new takeaways [65]. In addition, a third of surveyed UK LGAs had implemented planning restrictions on takeaway food outlets for reasons related to protecting the character of the area, which could also have positive health impacts [65]. To assist LGAs to implement action to address childhood obesity, the UK government has implemented a ‘trailblazer’ programme which provides funding and expert support to five projects, including a healthier retail programme which offers incentives, such as business support and subsidised advertising on LGA-owned properties, for food outlets that provide more healthy foods [66]. These actions provide a good starting point for LGAs in the EMHS area to consider.

### Strengths and limitations

This FODR assessment tool protocol is the first to rate the risk of food outlets to public health nutrition using objectively derived data, to the authors knowledge. Use of the FODR tool will assist in ensuring categorisation of dietary risk of specific food outlets is based on transparent and consistent criteria. In addition, the FODR assessment tool replicates the approach recommended by food regulators for risk assessment of food safety. There are limitations to its current use, which requires food outlet data to be collected for six public health nutrition attributes. Some of this data can be sourced using information obtained from the internet, including the food outlet websites and photographs posted to the Google search engine. The authors have provided two supplementary tables which include a summary of the literature describing public health nutrition impacts of food outlets (Supplementary Table 3) and Australian market data including details of major companies and their market share (Supplementary Table 4) which can also assist. Specific assessment of inappropriate marketing techniques such as making unhealthy foods appealing to children has not been factored into the FODR assessment. The ability of the FODR tool to incorporate the risk associated with on-site marketing will be assessed during pilot testing. Results from pilot testing and consultations with environmental health officers will inform future iterations of the FODR assessment tool, to ensure it provides a practical way of objectively evaluating the risk of food outlets to population diets.

## Conclusion

This protocol describes development of a food outlet risk assessment tool that incorporates six public health nutrition attributes. The risk assessment approach applied in this tool aims to assist LGAs to identify the healthfulness of food outlets and their potential impact on community food environments, and is the first to do so using objectively derived data. Although it was developed to assist the 13 LGAs within the EMHS area in Perth WA, the tool can also be applied throughout Australia and adapted for use in other countries. LGAs and public health practitioners will be able to use the FODR assessment tool to assist with making planning decisions, and to inform policies and interventions to improve the healthfulness of community food environments.

## Supplementary Information

**Additional file 1:** **Supplementary Table 1.** Completed SPIRIT 2013 Checklist: Recommended items to address in a clinical trial protocol and related documents*.

**Additional file 2:** **Supplementary Table 2.** Food business classification framework.

**Additional file 3:** **Supplementary Table 3.** Summary of public health nutrition impacts of consumer-facing food outlets.

**Additional file 4:** **Supplementary Table 4.** Market data to inform the public health nutrition risk rating tool for food outlets.

## Data Availability

The Food Outlet Dietary Risk Assessment Tool is provided in Fig. 1.

## Abbreviations

EMHS: East Metropolitan Health Service (in Perth, Western Australia)
FODR: Food Outlet Dietary Risk assessment tool
FSANZ: Food Standards Australia New Zealand
LGA: Local government authority
mRFEI: Modified Retail Food Environment Index
NEMS-R: Nutrition Environment Measures Survey for restaurants
NEMS-S: Nutrition Environment Measures Survey for supermarkets
UK: United Kingdom
WA: Western Australia
