# Supplementary material for: Food Outlets Dietary Risk (FODR) assessment tool: study protocol for assessing the public health nutrition risks of community food environments
Source: Nutr J. 2020 Nov 12;19:122. doi: 10.1186/s12937-020-00641-w (PMC7663896; doi:10.1186/s12937-020-00641-w)
Supplement: Supplementary file 2 — Additional file 2: Supplementary Table 2. Food business classification framework. [file 12937_2020_641_MOESM2_ESM.pdf]

Supplementary Table 2: Food business classification framework

Food retail	Food service	Charitable food provision	Food production and preparation	Institutional food
Supermarket	Café/coffee shop	Emergency food provision - groceries	Catering kitchens/home-based catering business/cooking classes	Hospitals
Discount grocery store	Restaurant	Emergency food provision - meals	Food truck/coffee or other drinks van/food market vendor	Residential care
Other grocery store	Fast casual/quick service/takeaway	Meals-on-wheels	Food home delivery service	Defence
Convenience store	Pub/tavern/bar/winery/distillery		Food manufacturer/processor	Correctional
World food/ethnic food store	Mobile/market/kiosk food		Packer, warehouse, food storage, food producer, wholesaler	Corporate (workplace)
Health food store	Entertainment venue			Education
Butcher/poultry store	Health and leisure venue			Childcare
Fishmonger	Accommodation with food			Community centre/church/hall/ function centre
Bakery				Residential worksite
Fruit and vegetable store/greengrocer				Other
Other specialist food retail outlet				
Liquor merchant/bottle shop				
General retail, not food or liquor				
