# Supplementary material for: Food Outlets Dietary Risk (FODR) assessment tool: study protocol for assessing the public health nutrition risks of community food environments
Source: Nutr J. 2020 Nov 12;19:122. doi: 10.1186/s12937-020-00641-w (PMC7663896; doi:10.1186/s12937-020-00641-w)
Supplement: Supplementary file 3 — Additional file 3: Supplementary Table 3. Summary of public health nutrition impacts of consumer-facing food outlets. [file 12937_2020_641_MOESM3_ESM.pdf]

Supplementary Table 3: Summary of public health nutrition impacts of consumer-facing food outlets

Food outlet classification	Facilitators and supports to healthy eating	Barriers and risks to healthy eating	Classified as healthy	Classified as unhealthy
**Supermarket**	Product: availability and variety<br>Availability of selected healthy foods was generally greater in chain supermarkets compared to independent supermarkets, in rural VIC (1).<br>Availability of selected healthy foods was generally greater in supermarkets compared to convenience stores, in rural NSW (2).<br>Supermarkets had better availability of fruit and vegetables than farmers' markets, in QLD (3).<br>Supermarkets had more fresh produce variety than other types of food outlets, in Brisbane (4).<br>Fresh fruit and vegetables contribute 12% of Australian supermarket sales (5).<br>US supermarkets protect food safety, and sell more variety of foods with higher nutritional quality at lower prices compared to other types of food outlets (6).<br>Product: nutritional quality<br>75% of Australian convenience foods were classified as healthy using nutrient profiling criteria (7).<br>Price<br>Food prices tended to be cheaper in supermarket chains compared to independent stores, in rural VIC (1, 8).	Product: availability<br>Woolworths has 50% market share of sales of all packaged alcohol in Australia (10).<br>57% of soft drink sales, and 48% of snack food sales are made in Australian supermarkets (5).<br>There was a large number of snack foods (1070) and drinks (863) in a Melbourne supermarket (11).<br>A third of Australian supermarket snacks were cakes, pies, sweet biscuits, and rich breads; 25% of snacks were available in multiple flavour varieties (11).<br>Product: nutritional quality<br>Only 9–22% of Australian snack foods and 14–27% of drinks could be classified as healthy in 2006 (11).<br>Less than one-third of Australian supermarket dairy, meat, and meat products were classified as healthy using Australian nutrient profiling criteria (7).<br>In Australia, two-thirds of child-orientated supermarket products had been reformulated between 2009 and 2011, however there was little overall improvement (12).<br>47% of Australian packaged foods were classified as healthy using nutrient profiling scoring criterion (7).	(20-33)	

Food outlet classification	Facilitators and supports to healthy eating	Barriers and risks to healthy eating	Classified as healthy	Classified as unhealthy
	Health outcomes<br><br>A systematic review of studies from the US and Canada concluded that a neighbourhood supermarket was more likely to be negatively associated with obesity rather than positively (9).	Placement<br><br>None of the Melbourne supermarkets surveyed displayed fruit at checkouts, and only one store displayed vegetables (13).<br><br>Most Australian supermarket checkouts displayed chocolate (87%), chewing gum (81%) and sweets (80%) (13).<br><br>Snack foods (crisps, chocolate, confectionery, soft drinks) were present at checkouts, island bins, and at a third of end-of-aisle displays in Australian supermarkets (14).<br><br>Snack foods (crisps, chocolate, confectionery, soft drinks) were displayed at 99% of Australian supermarket checkouts (15).<br><br>Chocolate was the most prominent snack foods on display in Australian supermarkets (14).<br><br>Promotion<br><br>75-82% of the products marketed to children via product packaging were unhealthy (16, 17).<br><br>Health outcomes<br><br>After opening a new supermarket in a UK food desert, diet diversity improved but poor dietary habits prevailed due to increased availability of nutrient-poor foods (18).		

Food outlet classification	Facilitators and supports to healthy eating	Barriers and risks to healthy eating	Classified as healthy	Classified as unhealthy
		<u>Health behaviour</u><br><br>Shopping primarily at supermarkets was not associated with a better nutrient profile of packaged foods purchased in the US (19).<br><br>74% of Australians purchased alcohol from a supermarket or stand-alone store owned by a supermarket group, compared to 11% from a hotel bottle shop, 10% from an independent bottle shop, and 4% from a wine club (10).		
**Discount grocery store**			(22)	
**Convenience store**		<u>Product: availability</u><br><br>Availability of selected healthy foods was generally greater in supermarkets compared to convenience stores in rural New South Wales (2).<br><br><u>Product: variety</u><br><br>Convenience stores had the least variety of fruit and vegetables compared to other types of food outlet in Brisbane (4).<br><br><u>Product: sales</u><br><br>38% of convenience store sales are from tobacco, 25% are from beverages, 11% are from snacks, confectionery and ice cream, 6% are from ready-to-eat food such as pies or muffins (34).	(20, 22, 28, 29)	(22-26, 30-33)

Food outlet classification	Facilitators and supports to healthy eating	Barriers and risks to healthy eating	Classified as healthy	Classified as unhealthy
		Placement<br>89% of products that children are exposed to at convenience store checkouts are unhealthy (35).		
**World food/ ethnic food store**			(22, 36)	
**Health food store**			(22-25, 33)	(29)
**Butcher**			(22, 26, 28)	(29) (23-25, 33)
**Fishmonger**			(22, 26, 28)	(29)
**Bakery**		Product: sales<br>Bread accounts for only 27% of sales made from bread and cake retail outlets (37).	(22, 28)	(22-26, 29, 33)
**Fruit and vegetables shop/ greengrocers/ farmer's market**		Product: availability<br>Farmers' markets had poorer availability of fruit and vegetables compared to supermarkets, in QLD (3).	(21-26, 29, 33)	
**Other specialist food outlet**				(22-26, 29, 33)
**Liquor merchant/ bottle shop**				(26, 29)
**General non-food**		Product: nutritional quality		(26, 29, 30)

Food outlet classification	Facilitators and supports to healthy eating	Barriers and risks to healthy eating	Classified as healthy	Classified as unhealthy
**retail outlet**		Most food and beverages available for sale in New York non-food outlets (e.g. auto shops, banks, clothing stores, hardware stores, gyms, laundromats, and salons) were less healthy (38).<br><br><u>Placement</u><br><br>16% of non-food stores present in a large UK shopping mall displayed unhealthy food at the checkout (39).		
**Café/ coffee shop**			(26, 30)	(28, 29)
**Restaurant**		<u>Product: nutritional quality</u><br><br>The energy content of main meals available at UK chain restaurants was excessive, and exceeded the energy content of fast food main meals (40).<br><br><u>Product: sales</u><br><br>Premium dining has the smallest number of outlets but generates the largest proportion of restaurant sales (39%) (41).<br><br>Low-cost restaurants, which offer simple value-based meals with few extras, have a higher amount of takeaway sales (41).<br><br>Steaks, burgers and general food, e.g. steaks, ribs, burgers, sandwiches, salads and fries accounted for 35% of sales from chain restaurants (42).<br><br><u>Product: food safety and quality</u>	(30)	(23-26, 29)

Food outlet classification	Facilitators and supports to healthy eating	Barriers and risks to healthy eating	Classified as healthy	Classified as unhealthy
		In the US, there were considerably more cases of illnesses and deaths due to poor food safety in restaurants compared to fast food outlets, particularly considering the number of meals served (FAO 2016).		
**Fast food/ QSR and takeaway**	<u>Product: nutritional quality</u><br><br>An Australian study identified "healthy" takeaway options as sandwiches, fruit and fruit products, fruit and/or vegetable juice, salads (except potato, pasta, rice salad), fried rice, diet soft drinks, coleslaw, pasta (43).<br><br>Presence of the following in takeaway or fast-food outlets was used to indicate a healthier takeaway food environment: fresh salad (not single toppings), alternatives with a high-fibre content (e.g. wholemeal or wholegrain bread, or a high proportion of vegetables), vegetable-based dishes (not deep-fried), and fresh fruit (however very few of these foods were present in the outlets surveyed) (2).<br><br><u>Product: sales</u><br><br>Sandwiches, salads and juices account for 15% of sales from fast food outlets (44).<br><br><u>Provision of nutritional information</u><br><br>In 2012, most fast food outlets (95%) provided some nutrition information but only 3% provided nutrition information for all menu items (45). Most (76%) nutrition information was accessible in 2010 (46), and almost all was accessible in 2012 (45).	<u>Product: nutritional quality</u><br><br>An Australian study identified "less healthy" takeaway options as chips, fries or wedges, soft drink, hamburger, pizza, cakes, sweet buns, muffins, scones, savoury pie, sausage roll, pastry, fried fish or seafood, ice cream, frozen yogurt, fried chicken, deep fried spring roll, dimsim or wonton, thick shake, milk shake (43).<br><br>Fast food is energy dense and nutrient poor compared to home prepared food (47).<br><br>Fast food portion sizes have increased over 50 years (48).<br><br>There are wide differences in the nutrient content of similar fast foods available in Australia (49).<br><br>Take-away food outlets had few healthy food items, in rural NSW (2).<br><br>Independent and smaller franchise fast food outlets may have higher energy content and use lower quality fats compared to chain fast food outlets (50).<br><br><u>Product: sales</u>		(20-33, 56, 57)

Food outlet classification	Facilitators and supports to healthy eating	Barriers and risks to healthy eating	Classified as healthy	Classified as unhealthy
		Burgers are the main products sold at fast food outlets (31%), followed by pizza (27%), and chicken-based fast food (18%) (44).<br><br>Single-serve chicken e.g. portions of chicken legs, breast, nuggets and burgers accounts for the largest share of sales from takeaway chicken shops (38%) (51).<br><br>Price<br><br>Energy-dense fast foods were cheaper per kilojoule than lower-energy density items. Salads had the highest energy cost, while value items, meals that included a dessert, and family meals had the lowest (52).<br><br>Provision of nutritional information<br><br>The total amount of information available increased over time, however fewer outlets provided nutrient values (45).<br><br>Health outcomes<br><br>Fast food leads to increased risk of obesity and type 2 diabetes (53).<br><br>Consumption of, and proximity to, fast food is established as a potential risk factor for diet-related health (54).<br><br>Neighbourhood fast food availability was positively associated with obesity (9).<br><br>There is an association between growth in fast food consumption and obesity in Australia (55).		

Food outlet classification	Facilitators and supports to healthy eating	Barriers and risks to healthy eating	Classified as healthy	Classified as unhealthy
**Pub/ Tavern/ Bar (licenced premises)**		Product: sales<br><br>On-premise sales of liquor accounted for 43% of sales, and meals and non-alcoholic beverages accounted for 8% (58).		(26, 29)
**Mobile, market and kiosk food**				(28, 30)
**Entertainment**				(30)
**Health and leisure**		Product: availability<br><br>Food and beverages available in sports venues and sports clubs tends to be energy-dense and nutrient-poor (59).<br><br>Promotion<br><br>Sponsorship of junior sport by food and beverage companies was found to be dominated by unhealthy foods (59).	(30)	

Footnote: NSW is New South Wales, VIC is Victoria, QLD is Queensland

References

1. Burns CM, Gibbon P, Boak R, Baudinette S, Dunbar JA. Food cost and availability in a rural setting in Australia. Rural Remote Health. 2004;4(4):311.
2. Innes-Hughes C, Boylan S, King LA, Lobb E. Measuring the food environment in three rural towns in New South Wales, Australia. Health Promotion Journal of Australia. 2012;23(2):129-33.
3. Millichamp A, Gallegos D. Comparing the availability, price, variety and quality of fruits and vegetables across retail outlets and by area-level socio-economic position. Public Health Nutr. 2013;16(1):171-8.
4. Winkler E, Turrell G, Patterson C. Does living in a disadvantaged area entail limited opportunities to purchase fresh fruit and vegetables in terms of price, availability, and variety? Findings from the Brisbane Food Study. Health Place. 2006;12(4):741-8.
5. Cloutman N. Supermarkets and grocery stores in Australia. IBISWorld Industry Report G4111. Australia: IBIS World; 2017. Available from: http://clients1.ibisworld.com.au.dbgw.lis.curtin.edu.au/reports/au/industry/default.aspx?entid=1834.
6. Stern D, Ng SW, Popkin BM. The Nutrient Content of U.S. Household Food Purchases by Store Type. Am J Prev Med. 2016;50(2):180-90.
7. Ni Mhurchu C, Brown R, Jiang Y, Eyles H, Dunford E, Neal B. Nutrient profile of 23 596 packaged supermarket foods and non-alcoholic beverages in Australia and New Zealand. Public Health Nutr. 2016;19(3):401-8.
8. Palermo CE, Walker KZ, Hill P, McDonald J. The cost of healthy food in rural Victoria. Rural Remote Health. 2008;8(4):1074.
9. Cobb LK, Appel LJ, Franco M, Jones-Smith JC, Nur A, Anderson CAM. The relationship of the local food environment with obesity: A systematic review of methods, study quality, and results. Obesity. 2015;23(7):1331-44.
10. Lucio R. Woolies now holds over 50 per cent of all alcohol sales Australia: Inside FMCG; 2018 [Available from: https://insidefmcg.com.au/2018/06/25/woolies-now-holds-over-50-per-cent-of-all-alcohol-sales/#daily.
11. Walker KZ, Woods JL, Rickard CA, Wong CK. Product variety in Australian snacks and drinks: how can the consumer make a healthy choice? Public Health Nutr. 2008;11(10):1046-53.
12. Savio S, Mehta K, Udell T, Coveney J. A survey of the reformulation of Australian child-oriented food products. BMC public health. 2013;13:836.
13. Dixon H, Scully M, Parkinson K. Pester power: snackfoods displayed at supermarket checkouts in Melbourne, Australia. Health Promotion Journal of Australia. 2006;17(2):124-7.
14. Thornton LE, Cameron AJ, McNaughton SA, Worsley A, Crawford DA. The availability of snack food displays that may trigger impulse purchases in Melbourne supermarkets. BMC public health. 2012;12:194.

15. Thornton LE, Cameron AJ, McNaughton SA, Waterlander WE, Sodergren M, Svastisalee C, et al. Does the availability of snack foods in supermarkets vary internationally? Int. 2013;10:56.
16. Chapman K, Nicholas P, Banovic D, Supramaniam R. The extent and nature of food promotion directed to children in Australian supermarkets. Health promotion international. 2006;21(4):331-9.
17. Mehta K, Phillips C, Ward P, Coveney J, Handsley E, Carter P. Marketing foods to children through product packaging: prolific, unhealthy and misleading. Public Health Nutr. 2012;15(9):1763-70.
18. Freire T, Rudkin S. Healthy food diversity and supermarket interventions: Evidence from the Seacroft Intervention Study. Food Policy. 2018.
19. Stern D, Poti JM, Ng SW, Robinson WR, Gordon-Larsen P, Popkin BM. Where people shop is not associated with the nutrient quality of packaged foods for any racial-ethnic group in the United States. The American Journal of Clinical Nutrition. 2016.
20. Pearce J, Blakely T, Witten K, Bartie P. Neighborhood Deprivation and Access to Fast-Food Retailing. Am J Prev Med. 2007;32(5):375-82.
21. Thornton LE, Crawford DA, Ball K. Neighbourhood-socioeconomic variation in women's diet: the role of nutrition environments. European journal of clinical nutrition. 2010;64(12):1423-32.
22. Thornton LE, Kavanagh AM. Association between fast food purchasing and the local food environment. Nutrition and Diabetes. 2012;2:e53.
23. Rundle A, Neckerman KM, Freeman L, Lovasi GS, Purciel M, Quinn J, et al. Neighborhood Food Environment and Walkability Predict Obesity in New York City. Environ Health Persp. 2009;117(3):442-7.
24. Vernez Moudon A, Drewnowski A, Duncan GE, Hurvitz PM, Saelens BE, Scharnhorst E. Characterizing the food environment: pitfalls and future directions. Public Health Nutr. 2013;16(7):1238-43.
25. Stark JH, Neckerman K, Lovasi GS, Konty K, Quinn J, Arno P, et al. Neighbourhood food environments and body mass index among New York City adults. J Epidemiol Commun H. 2013;67(9):736.
26. Moayyed H, Kelly B, Feng X, Flood V. Evaluation of a ‘healthiness’ rating system for food outlet types in Australian residential communities. Nutrition & Dietetics. 2017;74(1):29-35.
27. Rossimel A, Han SS, Larsen K, Palermo C. Access and affordability of nutritious food in metropolitan Melbourne. Nutrition & dietetics. 2014;73(1):13-8.
28. Larsen K, Cook B, Stone MR, Faulkner GEJ. Food access and children’s BMI in Toronto, Ontario: assessing how the food environment relates to overweight and obesity. Int J Public Health. 2015;60(1):69-77.
29. Lê Q, Nguyen HB, Terry DR, Dieters S, Auckland S, Long G. Quantifying and visualizing access to healthy food in a rural area of Australia: A spatial analysis. Food Sec. 2015;7(5):1017-29.

30. Tyrrell RL. Exploring adolescent food choice: a food environment perspective. UK: Newcastle University; 2014.

31. Engler-Stringer R, Shah T, Bell S, Muhajarine N. Geographic access to healthy and unhealthy food sources for children in neighbourhoods and from elementary schools in a mid-sized Canadian city. Spat Spatiotemporal Epidemiol. 2014;11:23-32.

32. Truong K, Fernandes M, An R, Shier V, Sturm R. Measuring the physical food environment and its relationship with obesity: Evidence from California. Public Health. 2010;124(2):115-8.

33. Janevic T, Borrell LN, Savitz DA, Herring AH, Rundle A. Neighbourhood food environment and gestational diabetes in New York City. Paediatric and Perinatal Epidemiology. 2010;24(3):249-54.

34. Magner L. Convenience stores in Australia. IBISWorld Industry Report G4112. Australia: IBISWorld; 2017. Available from: http://clients1.ibisworld.com.au.dbgw.lis.curtin.edu.au/reports/au/industry/default.aspx?entid=1835.

35. Horsley JA, Absalom KAR, Akiens EM, Dunk RJ, Ferguson AM. The proportion of unhealthy foodstuffs children are exposed to at the checkout of convenience supermarkets. Public Health Nutr. 2014;17(11):2453-8.

36. Spence JC, Cutumisu N, Edwards J, Raine KD, Smoyer-Tomic K. Relation between local food environments and obesity among adults. BMC public health. 2009;9:192.

37. Vuong B. Bread and cake retailing in Australia. IBISWorld Industry Report G4129. Australia: IBIS World; 2017. Available from: http://clients1.ibisworld.com.au.dbgw.lis.curtin.edu.au/reports/au/industry/default.aspx?entid=399.

38. Lucan SC, Maroko AR, Seitchik JL, Yoon DH, Sperry LE, Schechter CB. Unexpected Neighborhood Sources of Food and Drink: Implications for Research and Community Health. Am J Prev Med. 2018.

39. Wright J, Kamp E, White M, Adams J, Sowden S. Food at checkouts in non-food stores: a cross-sectional study of a large indoor shopping mall. Public Health Nutr. 2015;18(15):2786-93.

40. Robinson E, Jones A, Whitelock V, Mead BR, Haynes A. (Over)eating out at major UK restaurant chains: observational study of energy content of main meals. BMJ. 2018;363:k4982.

41. Magner L. Restaurants in Australia. IBISWorld Industry Report H4511a. Australia: IBISWorld; 2017. Available from: http://clients1.ibisworld.com.au.dbgw.lis.curtin.edu.au/reports/au/industry/default.aspx?entid=2010.

42. Magner L. Chain restaurants in Australia. IBISWorld Industry Report OD5489. Australia: IBISWorld; 2017. Available from: http://clients1.ibisworld.com.au.dbgw.lis.curtin.edu.au/reports/au/industry/default.aspx?entid=5489.

43. Miura K, Giskes K, Turrell G. Socioeconomic differences in takeaway food consumption and their contribution to inequalities in dietary intakes. J Epidemiol Commun H. 2009;63(10):820.

44. Magner L. Fast food services in Australia. IBISWorld Industry Report H4512. Australia: IBIS World; 2017.

45. Wellard L, Havill M, Hughes C, Watson WL, Chapman K. The availability and accessibility of nutrition information in fast food outlets in five states post-menu labelling legislation in New South Wales. Australian and New Zealand Journal of Public Health. 2015;39(6):546-9.

46. Wellard L, Glasson C, Chapman K, Miller C. Fast facts: The availability and accessibility of nutrition information in fast food chains. Health Promotion Journal of Australia. 2011;22(3):184-8.

47. Guthrie JF, Lin B-H, Frazao E. Role of Food Prepared Away from Home in the American Diet, 1977-78 versus 1994-96: Changes and Consequences. Journal of nutrition education and behavior. 2002;34(3):140-50.

48. Young LR, Nestle M. Expanding portion sizes in the US marketplace: Implications for nutrition counseling. Journal of the American Dietetic Association. 2003;103(2):231-40.

49. Dunford E, Webster J, Barzi F, Neal B. Nutrient content of products served by leading Australian fast food chains. Appetite. 2010;55(3):484-9.

50. Jaworowska A, Blackham T, Davies IG, Stevenson L. Nutritional challenges and health implications of takeaway and fast food. Nutr Rev. 2013;71(5):310-8.

51. Johnson S. Takeaway chicken shops in Australia. IBISWorld Industry Report OD5500. Australia: IBISWorld; 2017. Available from: http://clients1.ibisworld.com.au.dbgw.lis.curtin.edu.au/reports/au/industry/default.aspx?entid=5500.

52. Wellard L, Havill M, Hughes C, Watson WL, Chapman K. Energy-dense fast food products cost less: an observational study of the energy density and energy cost of Australian fast foods. Australian and New Zealand Journal of Public Health. 2015;39(6):544-5.

53. Jeffery RW, Baxter J, McGuire M, Linde J. Are fast food restaurants an environmental risk factor for obesity? Int J Behav Nutr Phy. 2006;3(1):2.

54. Thompson C, Ponsford R, Lewis D, Cummins S. Fast-food, everyday life and health: A qualitative study of ‘chicken shops’ in East London. Appetite. 2018;128:7-13.

55. Anaf J, Baum FE, Fisher M, Harris E, Friel S. Assessing the health impact of transnational corporations: a case study on McDonald’s Australia. Globalization Health. 2017;13(1):7.

56. Maguire ER, Burgoine T, Penney TL, Forouhi NG, Monsivais P. Does exposure to the food environment differ by socioeconomic position? Comparing area-based and person-centred metrics in the Fenland Study, UK. International Journal of Health Geographics. 2017;16(1):33.

57. Maguire ER, Burgoine T, Monsivais P. Area deprivation and the food environment over time: A repeated cross-sectional study on takeaway outlet density and supermarket presence in Norfolk, UK, 1990–2008. Health Place. 2015;33:142-7.

58. McGregor W. Pubs, bars and nightclubs in Australia. IBISWorld Industry Report H4520. Australia: IBISWorld; 2017. Available from: http://clients1.ibisworld.com.au.dbgw.lis.curtin.edu.au/reports/au/industry/default.aspx?entid=448.

59. Carter M-A, Edwards R, Signal L, Hoek J. Availability and marketing of food and beverages to children through sports settings: a systematic review. Public Health Nutr. 2011;15(8):1373-9.
