# Supplementary material for: Food Outlets Dietary Risk (FODR) assessment tool: study protocol for assessing the public health nutrition risks of community food environments
Source: Nutr J. 2020 Nov 12;19:122. doi: 10.1186/s12937-020-00641-w (PMC7663896; doi:10.1186/s12937-020-00641-w)
Supplement: Supplementary file 4 — Additional file 4: Supplementary Table 4. Market data to inform the public health nutrition risk rating tool for food outlets. [file 12937_2020_641_MOESM4_ESM.pdf]

Supplementary Table 4: Market data to inform the public health nutrition risk rating tool for food outlets

IBISWorld Industry Report	Industry definition	Revenue 2016-17	Number of businesses	Key players	Key brands	Major companies	Company % share	Products	Product % share
**Food retail outlets**									
Supermarkets, grocery stores, and discount grocery stores (1)	Establishments that sell a range of groceries and food products including fruit, vegetables, bread, cigarettes, canned goods, toiletries, and delicatessen items	$105.3bn	2,012	Woolworths, Wesfarmers (Coles), Aldi, Metcash (IGA)	Woolworths, Coles, Aldi, IGA	Woolworths	33.6	Dry and packaged foods	28.5
						Wesfarmers owns Coles and Bi-Lo	29.3	Milk and other dairy products	13.3
						ALDI Stores Supermarkets	8.9	Fresh fruit and vegetables	12.1
						Metcash owns the IGA, SupaIGA, IGA X-press, Foodland brands which operate under franchise arrangements	7.1	Toiletries and health products	10.7
								Cigarettes and general merchandise e.g. stationery, containers, pet food	10.5
								Meat products	9.9
								Beverages	8.2
								Bread and bakery products	6.8
Convenience stores (2)	Establishments that sell a range of products including cigarettes, beverages, confectionery, snack foods, ready-to-eat foods, mobile phones, newspapers and general merchandise. Includes milk bars, corner stores, stores attached to fuel stations	$4.6bn	8,180	Metcash and 7-Eleven	Lucky 7, 7-Eleven	Metcash operates Lucky 7	12.2	Tobacco	37.6
						7-Eleven Stores	7.4	Beverages	25.3
								Snacks, confectionery, ice cream	10.7
								Communications, travel and car accessories e.g. phone cards, tickets, travel cards	10.1
								Groceries e.g. tinned food, bread, instant noodles	7.4
								Ready-to-eat food e.g. sandwiches, muffins, pies, chips	5.7
Fresh meat, fish and poultry retailing (3)	Establishments that primarily retail fresh meat, fish and poultry including specialist retailers such as butchers and fishmongers	$6.9bn	4,646	No major players	Lenard's	Lenard's	3.1	Fresh beef and veal	38.9
						Tasman Market Fresh Meats	2.2	Fresh poultry	29.3
								Fresh lamb	12.4
								Fresh pork	9.9
								Fresh seafood	8.5
								Other fresh meats e.g. kangaroo, venison, rabbit	1.0
Fruit and vegetable retailing (4)	Establishments that primarily retail fresh fruit and vegetables	$3.7bn	2,718	No major players	none	Large number of small retailers	n/a	Other vegetables e.g. beans, capsicum, carrots, lettuce, mushrooms, onions	40.2
								Other fruit e.g. oranges, mandarins, strawberries, pineapples	16.1
								Apples and pears	12.1
								Stone fruit	11.0
								Potatoes	10.4
								Bananas	6.3
								Tomatoes	3.9
Bread and cake retailing (5)	Establishments that sell a range of baked goods including bread, cakes, pastries and biscuits either independently or as part of a franchise. Products are made off-premises	$760.3m	745	Most operators are small-scale firms	Donut King, Krispy Kreme	Retail Food Group operate Donut King	21.6	Cakes	39.1
						Breadtop	7.6	Pastries and other bakery products e.g. pies, sausage rolls, quiche, tarts, Danish pastries, muffins, biscuits, cookies, doughnuts	33.3
						KKA Holdings operates Krispy Kreme Doughnuts	1.0	Bread including loaves, rolls, savoury buns	26.6
Liquor merchant or bottle shop (6)	Establishments that sell beer, wine, spirits, ready-to-drink mixers and other alcohol in packaged form for consumption away from the premises	$11.7bn	2,371	Woolworths, Wesfarmers (Coles)	Dan Murphy's, BWS, Woolworths Liquor, Liquorland, Vintage Cellars, Fist Choice Liquor	Woolworths	45.4	Beer	39.9
						Coles	18.6	Wine	37.4
						Independent Brands Australia	9.0	Bottled spirits	12.9
						ALDI Stores Supermarkets	3.5	Ready-mixed-drinks	6.0
								Cider	3.8

IBISWorld Industry Report	Industry definition	Revenue 2016-17	Number of businesses	Key players	Key brands	Major companies	Company % share	Products	Product % share
**Food service outlets**									
Cafes and coffee shops (7)	Establishments selling food and beverages to customers on the premises. Coffee shops tend to sell a larger range of sweet foods, whereas cafes tend to sell a more extensive range of sweet and savoury items (classified as restaurants if also sell dinner)	$8.2bn	14,071	No major players	Michel's Patisserie, Gloria Jean's	Retail Food Group operate Michel's Patisserie, bb's café, Esquires Coffee, Gloria Jean's Coffees	4.8	Coffee	51.0
						Minor DKL Food Group franchise The Coffee Club and Coffee Hit	1.6	Food including breakfast and lunch items e.g. eggs, toast, sandwiches, muffins, cakes, cookies	27.5
						Emirates Leisure Retail Australia franchise Hudsons Coffee chain	1.0	Other beverages e.g. milkshakes, tea, hot chocolate, chai, water, juice, soft drinks	21.5
						Starbuck's Coffee Australia	1.0		
Fast food services (8)	Establishments that provide fast food including burgers, pizza, sandwiches, sushi, to takeaway for immediate consumption. Food is provided in takeaway containers but some is consumed on premise, including in food halls or food courts	$19.3bn	24,893	McDonalds, Yum! Restaurants, Subway Systems Australia	McDonald's, Hungry Jack's, Pizza Hut, Domino's	McDonald's Australia	20.2	Burgers	31.4
						Yum! Restaurants operates KFC and Pizza Hut	7.8	Pizza	26.6
						Subway Systems Australia operates Subway	7.4	Chicken based fast food	17.5
						Competitive Foods Australia operate Hungry Jack's	7.1	Sandwiches, salads, juices	15.1
						Domino's Pizza Enterprises operate Domino's	4.9	Other e.g. pies, sausage rolls, fish and chips, Thai, Japanese, Indian, Chinese and Mexican fast food	4.8
						Quick Service Restaurant Group operates Red Rooster, Oporto, Chicken Treat	4.2	Desserts and confectionery	4.6
						Collins Foods operates KFC (including in WA)	3.1		
						Retail Zoo operates Boost Juice, Salsa's Fresh Mex Grill and Hatch Chicken Shop	2.4		
Fast food burger shops (9)	Establishments that sell burgers for consumption on premises or takeaway	$7.0bn	5,910	Two main companies with the remainder being a multitude of niche burger shops	McDonald's, Hungry Jack's	McDonald's Australia	56.5	Burgers	51.1
						Competitive Foods Australia operate Hungry Jack's	19.9	Other products e.g. wraps, salads, desserts	17.7
						Grill'd	4.6	Breakfast food e.g. pancakes, hash browns, breakfast wraps and burgers	12.4
						Burger Edge	1.0	Beverages e.g. soft drinks, juices, milk shakes, coffee, alcohol	10.8
								Sides e.g. fries, onion rings, potato salad	8.0
Pizza restaurants and takeaway (10)	Establishments that primarily sell traditional and gourmet pizzas. Pizzas can be ordered in advance, to take away or eat on the premises.	$3.7bn	4,004	Most of the industry is independently owned outlets	Domino's, Pizza Hut, Crust Gourmet Pizza, Pizza Capers, Eagle Boys	Domino's Pizza Enterprises operate Domino's	25.6	Traditional pizzas	42.7
						Out of the Box Group operated Pizza Hut	15.7	Gourmet and specialty pizzas, often made with more expensive or imported ingredients	22.6
						Retail Food Group operate Pizza Capers and Crust Gourmet Pizza	4.0	Pizza sides e.g. garlic bread, chips, wedges, chicken wings, beef ribs, dips and sauces	18.3
						La Porchetta Holdings operates full service restaurants	1.9	Other products including desserts and beverages	16.4
Restaurants (11)	Establishments that sell food and beverages for consumption on the premises. Customers generally order, are served, and pay after eating. Theatre and chain restaurants are excluded.	$20.4bn	26,377	No major players	None	None, most restaurants are owner-operated	n/a	Premium dining, the smallest number of outlets which generates the largest proportion of sales	39.0
								Mid-range dining, with fashionable new restaurants often falling into this category	35.0
								Low-cost dining, which offer simple value-based meals with few extras, and have a higher amount of takeaway sales	26.0

IBISWorld Industry Report	Industry definition	Revenue 2016-17	Number of businesses	Key players	Key brands	Major companies	Company % share	Products	Product % share
Chain restaurants (12)	Establishments that have seating and provide table service, with outlets in at least 3 locations. BYO, licenced and unlicensed restaurants are included.	$389.0m	30	A variety of chain restaurants specialise in a wide range of cuisines	La Porchetta, Fasta Pasta, TGI Fridays, Hog's Breath Café, Outback Steakhouse, Taco Bill, Amigos, China Bar, Dragon Palace, Pancake Parlour	Hog's Breath Café operates a chain of steakhouse restaurants	20.5	Italian food from chains such as La Porchetta and Fasta Pasta, with a focus on pizza and pasta	40.1
						La Porchetta Holdings operates full service restaurants	12.7	Steaks, burgers and general food, e.g. steaks, ribs, burgers, sandwiches, salads and fries	34.5
						Taco Bill Mexican has more than 30 franchise-operated restaurants	6.9	Mexican food e.g. burritos, tortillas, quesadillas, and fajitas	12.2
						Fasta Pasta	5.0	Asian food	9.3
						Lovely Pancakes operates 12 Pancake Parlour outlets	4.0	Breakfast food e.g. pancakes, crepes	3.9
Takeaway chicken shops (13)	Establishments that primarily sell chicken and chicken-based foods for on premise and takeaway consumption	$3.4bn	2,154	Large fast food chains dominate	KFC, Red Rooster, Nandos, Oporto, Chicken Treat	Yum! Restaurants operates KFC	36.2	Single-serve chicken e.g. portions of chicken legs, breast, nuggets and burgers for one consumer - accounts for the largest share of sales	38.0
						Quick Service Restaurant Group operates Red Rooster, Oporto, Chicken Treat	20.4	Family-serve chicken e.g. family-sized portions of chicken legs, breast, nuggets and burgers for a group of consumers	21.2
						Collins Foods operates KFC (including in WA)	17.1	Other food e.g. chips, salads, potato and gravy, bread, rice, hash browns - more important in small independents than chains	19.8
						Nando's Australia operates Nando's	9.8	Beverages e.g. soft drinks, juice, water	12.7
								Desserts - account for the smallest share of sales	8.3
Fish and chip shops (14)	Establishments that supply fish and chips for immediate consumption. Customers order and pay prior to eating. Food is provided in takeaway containers to take away or eat on the premises.	$657.9m	3,415	none	none	There are no major players, with most being owner-operators	n/a	Fish and seafood	35.5
								Burgers and other food e.g. potato cakes, dim sim, souvlakis, sandwiches, salads and snacks	32.9
								Chips	24.6
								Beverages e.g. soft drinks	7.0
Pubs, bars and nightclubs (15)	Establishments that sell alcohol for consumption on premises (e.g. bars) or off the premises (e.g. drive-through bottle shops), and sometimes provide food service, entertainment and gambling	$17.2bn	5,906	Woolworths	none	Woolworths owns pubs, has 75% equity in a hotel group, and is one of the biggest poker machine operators. Brands include Dan Murphy's and BWS	9.2	Liquor for on premise consumption (nightclubs account for a large proportion of this segment)	43.0
						Wesfarmers owns and operates hotels, has poker machines and owns liquor retail outlets	2.4	Gaming and wagering	22.0
						Merivale Group operated pubs, bars, hotels and restaurants in Sydney	1.0	Liquor for off-premise consumption from bottle shops that are attached to bars and pubs owned by Coles and Woolworths	21.0
						Independent Pub Group Holdings owns hotels and liquor retail outlets	1.0	Meals and non-alcoholic beverages for consumption on the premises e.g. gastropubs	8.0
								Other products and services e.g. sales of merchandise, entrance fees	6.0

References

1. Cloutman N. Supermarkets and grocery stores in Australia. IBISWorld Industry Report G4111. Australia: IBIS World; 2017. Available from: http://clients1.ibisworld.com.au.dbgw.lis.curtin.edu.au/reports/au/industry/default.aspx?entid=1834.
2. Magner L. Convenience stores in Australia. IBISWorld Industry Report G4112. Australia: IBISWorld; 2017. Available from: http://clients1.ibisworld.com.au.dbgw.lis.curtin.edu.au/reports/au/industry/default.aspx?entid=1835.
3. Cloutman N. Fresh meat, fish and poultry retailing in Australia. IBISWorld Industry Report G4121. Australia: IBISWorld; 2017. Available from: http://clients1.ibisworld.com.au.dbgw.lis.curtin.edu.au/reports/au/industry/default.aspx?entid=396.
4. Vuong B. Fruit and vegetable retailing in Australia. IBISWorld Industry Report G4122. Australia: IBISWorld; 2017. Available from: http://clients1.ibisworld.com.au.dbgw.lis.curtin.edu.au/reports/au/industry/default.aspx?entid=397.
5. Vuong B. Bread and cake retailing in Australia. IBISWorld Industry Report G4129. Australia: IBIS World; 2017. Available from: http://clients1.ibisworld.com.au.dbgw.lis.curtin.edu.au/reports/au/industry/default.aspx?entid=399.
6. Thomson J. Liquor retailing in Australia. IBISWorld Industry Report G4123. Australia: IBISWorld; 2018.
7. Vuong B. Cafes and coffee shops in Australia. IBISWorld Industry Report H4511b. Australia: IBIS World; 2017. Available from: http://clients1.ibisworld.com.au.dbgw.lis.curtin.edu.au/reports/au/industry/default.aspx?entid=2015.
8. Magner L. Fast food services in Australia. IBISWorld Industry Report H4512. Australia: IBIS World; 2017.
9. Magner L. Fast food burger shops in Australia. IBISWorld Industry Report OD5498. Australia: IBISWorld; 2017. Available from: http://clients1.ibisworld.com.au.dbgw.lis.curtin.edu.au/reports/au/industry/default.aspx?entid=5498.
10. Vuong B. Pizza restaurants and takeaway in Australia. IBISWorld Industry Report OD4025. Australia: IBISWorld; 2017. Available from: http://clients1.ibisworld.com.au.dbgw.lis.curtin.edu.au/reports/au/industry/default.aspx?entid=4025.
11. Magner L. Restaurants in Australia. IBISWorld Industry Report H4511a. Australia: IBISWorld; 2017. Available from: http://clients1.ibisworld.com.au.dbgw.lis.curtin.edu.au/reports/au/industry/default.aspx?entid=2010.
12. Magner L. Chain restaurants in Australia. IBISWorld Industry Report OD5489. Australia: IBISWorld; 2017. Available from: http://clients1.ibisworld.com.au.dbgw.lis.curtin.edu.au/reports/au/industry/default.aspx?entid=5489.
13. Johnson S. Takeaway chicken shops in Australia. IBISWorld Industry Report OD5500. Australia: IBISWorld; 2017. Available from: http://clients1.ibisworld.com.au.dbgw.lis.curtin.edu.au/reports/au/industry/default.aspx?entid=5500.
14. Vuong B. Fish and chip shops in Australia. IBISWorld Industry Report OD5499. Australia: IBISWorld; 2017. Available from: http://clients1.ibisworld.com.au.dbgw.lis.curtin.edu.au/reports/au/industry/default.aspx?entid=5499.
15. McGregor W. Pubs, bars and nightclubs in Australia. IBISWorld Industry Report H4520. Australia: IBISWorld; 2017. Available from: http://clients1.ibisworld.com.au.dbgw.lis.curtin.edu.au/reports/au/industry/default.aspx?entid=448.
